# The Effectiveness of Fully Automated Digital Interventions in Promoting Mental Well-Being in the General Population: Systematic Review and Meta-Analysis

**DOI:** 10.2196/44658

**Published:** 2023-10-19

**Authors:** Julia Groot, Alexander MacLellan, Madelaine Butler, Elisa Todor, Mahnoor Zulfiqar, Timothy Thackrah, Christopher Clarke, Mark Brosnan, Ben Ainsworth

**Affiliations:** 1 Department of Psychology University of Bath Bath United Kingdom; 2 Cyberlimbic Systems Ltd London United Kingdom; 3 Department of Computer Science University of Bath Bath United Kingdom; 4 Centre for Applied Autism Research Department of Psychology University of Bath Bath United Kingdom; 5 School of Psychology Faculty of Environmental and Life Sciences University of Southampton Southampton United Kingdom

**Keywords:** mental well-being, promotion, intervention, digital, web-based, apps, mobile phone

## Abstract

**Background:**

Recent years have highlighted an increasing need to promote mental well-being in the general population. This has led to a rapidly growing market for fully automated digital mental well-being tools. Although many individuals have started using these tools in their daily lives, evidence on the overall effectiveness of digital mental well-being tools is currently lacking.

**Objective:**

This study aims to review the evidence on the effectiveness of fully automated digital interventions in promoting mental well-being in the general population.

**Methods:**

Following the preregistration of the systematic review protocol on PROSPERO, searches were carried out in MEDLINE, Web of Science, Cochrane, PsycINFO, PsycEXTRA, Scopus, and ACM Digital (initial searches in February 2022; updated in October 2022). Studies were included if they contained a general population sample and a fully automated digital intervention that exclusively used psychological mental well-being promotion activities. Two reviewers, blinded to each other’s decisions, conducted data selection, extraction, and quality assessment of the included studies. Narrative synthesis and a random-effects model of per-protocol data were adopted.

**Results:**

We included 19 studies that involved 7243 participants. These studies included 24 fully automated digital mental well-being interventions, of which 15 (63%) were included in the meta-analysis. Compared with no intervention, there was a significant small effect of fully automated digital mental well-being interventions on mental well-being in the general population (standardized mean difference 0.19, 95% CI 0.04**-**0.33; *P*=.02). Specifically, mindfulness-, acceptance-, commitment-, and compassion-based interventions significantly promoted mental well-being in the general population (*P*=.006); insufficient evidence was available for positive psychology and cognitive behavioral therapy**–**based interventions; and contraindications were found for integrative approaches. Overall, there was substantial heterogeneity, which could be partially explained by the intervention duration, comparator, and study outcomes. The risk of bias was high, and confidence in the quality of the evidence was very low (Grading of Recommendations, Assessment, Development, and Evaluations), primarily because of the high rates of study dropout (average 37%; range 0%-85%) and suboptimal intervention adherence (average 40%).

**Conclusions:**

This study provides a novel contribution to knowledge regarding the effectiveness, strengths, and weaknesses of fully automated digital mental well-being interventions in the general population. Future research and practice should consider these findings when developing fully automated digital mental well-being tools. In addition, research should aim to investigate positive psychology and cognitive behavioral therapy–based tools as well as develop further strategies to improve adherence and reduce dropout in fully automated digital mental well-being interventions. Finally, it should aim to understand when and for whom these interventions are particularly beneficial.

**Trial Registration:**

PROSPERO CRD42022310702; https://tinyurl.com/yc7tcwy7

## Introduction

### General Background

Mental well-being is commonly defined as a complex construct that includes a subjective experience (subjective well-being, which is often referred to as “happiness”) [1] and a process of self-realization (psychological well-being) [2,3]. Traditionally, it was thought that mental well-being would arise in the absence of mental illness, as they were considered opposite ends of 1 continuum [4]. However, the absence of mental illness was found to be insufficient to produce good mental well-being [5]. The dual-continuum model has identified that mental well-being and mental illness are 2 distinct but related continua instead [6], both of which could be considered part of mental health [7]. It is important to focus exclusively on the effective promotion of mental well-being [8], as only a small proportion of the general population has optimal levels of mental well-being [7,9].

In addition, mental well-being in the general population is crucial for allowing society and the individuals within it to thrive. Improved mental well-being is connected to increased productivity, personal growth, a higher quality of life, stronger social cohesion, and more fulfilling and lasting relationships, as well as a decreased likelihood of developing diseases and mental illnesses and a longer lifespan [5,7,10,11]. Promoting mental well-being in the general population is therefore considered a fundamental goal by the World Health Organization (WHO), as described in the Mental Health Action Plan 2013-2030 [12]. Mental well-being promotion interventions provide “various activities or practices that aim to promote, build on, increase or foster primarily individuals’ strengths, resourcefulness or resiliency” [10].

Evidence suggests that a variety of psychological approaches are effective in promoting mental well-being, including acceptance and commitment therapy (ACT), compassion, cognitive behavioral therapy (CBT), mindfulness, positive psychology, and multitheoretical interventions [7]. These psychological approaches were found to have small to moderate effects on mental well-being in the general population, whereby mindfulness-based interventions (MBIs) and multicomponent positive psychology interventions were particularly efficacious [7,13]. Further meta-analyses focusing on positive psychology interventions, MBIs, and ACT-based interventions separately also found similar effects on mental well-being [14-16].

However, these systematic reviews did not focus on fully automated digital interventions. Fully automated digital interventions are interventions that are delivered entirely by the technology itself, not requiring any form of human support (by clinicians or nonclinicians) [17]. Although fully automated digital interventions might be less effective, as recent research has found that any form of human support enhances the effectiveness of interventions [18], fully automated digital interventions allow for great scalability and are highly cost-effective and accessible [19]. Therefore, fully automated digital interventions provide a particularly pertinent way to promote mental well-being in the general population.

Overall, there is a need to systematically review the evidence of the effectiveness of fully automated digital mental well-being interventions to improve mental well-being (which includes subjective and psychological well-being) in the general population. Furthermore, an understanding of what psychological approaches work when delivered fully automated digitally and for whom (as one approach does not suit all) [20] is needed.

### Main Objective

This systematic review aims to understand the effectiveness of fully automated digital interventions in promoting mental well-being in the general population.

### Secondary Objectives

Furthermore, the systematic review aims to explore the effectiveness of fully automated digital mental well-being interventions across psychological approaches and population subgroups.

## Methods

### Study Protocol

The systematic review protocol was registered on PROSPERO (CRD42022310702). The Cochrane handbook was used when designing and conducting the systematic review [21], and PRISMA (Preferred Reporting Items for Systematic Reviews and Meta-Analyses) guidelines were followed for reporting of the systematic review [22].

### Eligibility Criteria

Studies were included if they used a fully automated digital intervention that aimed to promote mental well-being in the general population.

The study needed to include adults, meaning that the population needed to be aged ≥18 years. General population was further defined as any adult population subgroup that was not a clinical population and was not specifically recruited by the researchers because of (expected) lower mental well-being baseline scores.

Digital interventions were defined according to the National Institute for Health and Care Excellence [17] as interventions that are delivered through hardware and electronic devices (eg, smartwatches and smartphones), software (eg, computer programs and apps), and websites. The intervention needed to be fully automated, which means it should be delivered by the technology itself entirely, independent from health care professionals, and not containing any other form of social support [17]. For example, a digital web-based intervention in which video content was delivered automatically would have been included, whereas a digital video call intervention in which a health care professional delivered content would have been excluded. Although the content should be delivered entirely by the technology itself, the elements of the study could still have been conducted by the researchers. For example, researchers could have screened, obtained measures, and obtained informed consent (digitally or in person), after which they could have provided the participants with access to the intervention.

Furthermore, the intervention needed to use individual mental well-being promotion, defined by the WHO as “various activities or practices that aim to promote, build on, increase or foster primarily individuals’ strengths, resourcefulness or resiliency” [10]. This should be a psychological intervention.

Interventions that included physical activity–related or lifestyle-related interventions were excluded. If an intervention contained elements that did not include mental well-being promotion, they would also be excluded, as the detection of the effectiveness of mental well-being promotion strategies would not be possible. For example, an MBI would have been included; however, an MBI that included a yoga session would have been excluded.

The outcome needed to consider a validated measure of mental well-being, including psychological well-being or subjective well-being.

Finally, studies needed to investigate the effectiveness of this digital intervention on mental well-being. Therefore, quantitative randomized and nonrandomized studies of interventions, such as before-after studies, were considered appropriate, as they can provide insights into the effectiveness of interventions [23]. For further details regarding the inclusion and exclusion criteria, please refer to the protocol [24].

### Searches

The initial search was conducted in February 2022 and updated using a title and keyword search in October 2022. The databases searched included MEDLINE, Web of Science, Cochrane, PsycINFO, PsycEXTRA, Scopus, and ACM Digital. Combinations of the following key search terms were used: “mental well being,” “mental wellbeing,” “psychological well being,” “psychological wellbeing,” “subjective well being,” and “subjective wellbeing,” in combination with “digital*,” “online,” “internet,” “web-based,” “app,” “apps,” “smartphone application*,” and “mobile application*.” No restrictions were applied. Refer to Multimedia Appendix 1 [25-42] for the detailed searches conducted in each database.

### Study Selection

Each record was double screened, and the reviewers were blinded to each other’s decisions throughout the process. To ensure consistency and quality of the screening process, the lead author (JG) screened all records, and double screening was conducted by MB, ET, and MZ. After screening 10.71% (776/7764) of the records, interreviewer reliability was calculated, which ranged from moderate to substantial agreement (Cohen κ=0.54-0.79) [43]. Inconsistencies in the screening process were discussed, and conflicts were resolved through discussion. If conflicts remained, an additional discussion with a third, senior reviewer (BA) was conducted. Upon completion of the screening, interreviewer reliability was recalculated (Cohen κ=0.42-0.80), and conflicts were again resolved using the same process. This process was then repeated for full-text screening.

### Data Extraction

Before data extraction, the Cochrane data collection form was adapted and prepiloted for this review. Data extraction included information regarding the study population, participant demographics, and setting; details of the intervention and control conditions (such as duration, frequency, timing, and activities); study methodology; recruitment and study completion rates; outcomes, outcome measures, and times of measurement; and information for the assessment of the risk of bias (RoB). Two reviewers (JG and AM) independently extracted all relevant data from the included studies and held meetings to discuss any discrepancies in data extraction. When conflicting views on the data extraction occurred, a third, senior reviewer (BA) advised on how to resolve the issue. Missing data were sought by contacting the lead author of the study via email, which was identified through the journal paper.

### RoB Assessment

RoB was assessed independently by 2 reviewers (JG and AM) using the Cochrane RoB 2.0 tool for randomized controlled trials (RCTs) [44]. No standardized tools were available for noncontrolled before-after studies; therefore, the National Institutes of Health tool, “Quality Assessment Tool for Before-After (Pre-Post) Studies with No Control Group,” was used as a guidance to provide an indication of the RoB in these studies [45]. However, it was considered that these studies would provide a lower quality of evidence. Following the RoB assessments, discussions were held to discuss conflicts, and any remaining disagreements were resolved through verbal discussion with a third reviewer (BA).

### Data Synthesis and Meta-Analysis

Mean, SD, and total number of participants were extracted for each postintervention mental well-being outcome in the study arms that met the inclusion criteria of the digital mental well-being intervention and control group. The effect estimates were averaged, where the studies included multiple study outcomes. This method was also adopted for multiarm studies because it was considered meaningful to combine the intervention effects, as all the included intervention arms were digital mental well-being interventions. In addition, this avoided double counting of participants in the control group. Standardized mean differences (SMDs) were used in a random-effects model.

Initially, both the per-protocol (PP) and intention to treat (ITT) data were extracted. However, only PP data were included in the meta-analysis, as high dropout rates (ranging up to 85%) led to ITT data being less meaningful.

Visual inspection of the forest plot and the chi-square and *I*^2^ tests were used to assess heterogeneity. A value of >50% was considered to represent substantial heterogeneity. Heterogeneity was explored, interpreted, and contextualized.

## Results

### Description of Studies

**Figure 1:**
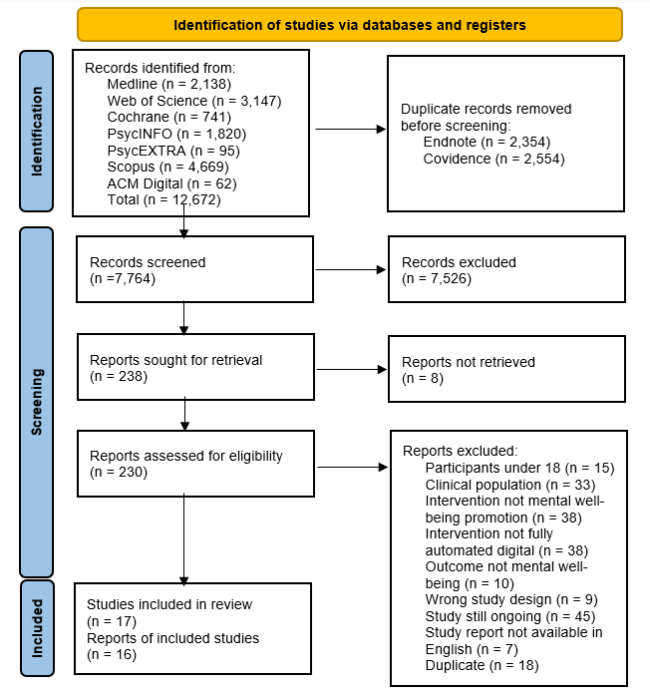
PRISMA (Preferred Reporting Items for Systematic Reviews and Meta-Analyses) flowchart of the search strategy outcomes.

An initial search yielded 12,672 records. Following deduplication, 7764 records were screened in Covidence (Veritas Health Innovation). A total of 7526 records were excluded following title and abstract screening, and 238 records were sought for retrieval for full-text screening. A total of 230 full-text records were screened, leading to the exclusion of another 213 records. The most common reasons for exclusion were the population being a clinical population, intervention not solely using mental well-being promotion, intervention not being fully automated and digital, or that the study was still ongoing. For full details of the study selection process, refer to Figure 1.

An updated title and keyword search in October 2022 yielded another 525 records. After deduplication, 366 articles were screened in Covidence. A total of 347 articles were excluded, and full texts of 19 articles were obtained. Furthermore, 17 articles were excluded following full-text screening.

### Narrative Summary

A total of 18 records containing 19 studies were included in this systematic review, including 17 RCTs and 2 non-RCTs before-after trials.

### Setting and Participants

Studies mainly occurred in Western countries; the participants were primarily female and highly educated; and the study populations were students, employees, mothers, and other general population samples (Table 1).

**Table 1:** Characteristics of the included studies.

Study, year	Population	Setting	Comparator	Outcome^a^
Study 3 from Avey et al [25], 2022	Employees	United States and Australia	Unknown	PWB^b^
Bakker et al [26], 2018	General population	Australia	Waitlist control	MWB^c^
Brazier et al [27], 2022	Trainees	United Kingdom	Waitlist control	MWB
Champion et al [28], 2018	Employees	United Kingdom and United States	Waitlist control	SWB^d^
Chung et al [29], 2021	Students	Australia and United Kingdom	Waitlist control	MWB
Study 1 from Di Consiglio et al [30], 2021	Students	Italy	Active control	PWB
Study 2 from Di Consiglio et al [30], 2021	Students	Italy	None	PWB
Eisenstadt et al [31], 2021	Real-world app users	United Kingdom	None	MWB
Gammer et al [32], 2020	Mothers of infants aged <1 y	United Kingdom	Waitlist control	MWB
Liu et al [33], 2021	Students	China	Placebo	SWB
Ly et al [34], 2017	General population	Sweden	Waitlist control	PWB and SWB
Mak et al [35], 2018	General population	China	Active control	MWB
Manthey et al [36], 2016	General population	Germany	Active control	SWB
Mitchell et al [37], 2009	Adults	Australia	Placebo	PWB
Neumeier et al [38], 2017	Employees	Germany and Australia	Waitlist control	SWB
Pheh et al [39], 2020	General population	Malaysia	Active control	MWB
Schulte-Frankenfeld and Trautwein [40], 2021	Students with a part-time job	Germany	Waitlist control	SWB
Shin et al [41], 2020	Students	United States	Placebo	SWB
Walsh et al [42], 2019	Students	Canada	Active control	PWB

^a^Mental well-being outcomes included 5-item mental well-being index (World Health Organization-5) [46] and Warwick-Edinburgh Mental Well-Being Scale (version 1) [47]. Subjective well-being outcomes included Satisfaction With Life Scale [48], Positive And Negative Affect Schedule [49], Satisfaction with Life and happiness [50], Subjective Happiness Scale [51], and single-item life satisfaction and affect measure [38]. Psychological well-being outcomes included psychological well-being [52], Psychological Well-Being Scale [53], Psychological Well-Being Index (adult) scale [54], and Flourishing Scale [34].

^b^PWB: psychological well-being.

^c^MWB: mental well-being.

^d^SWB: subjective well-being.

### Psychological Approaches

Several different psychological approaches were used, including the following: (1) mindfulness, ACT, and self-compassion; (2) positive psychology; (3) cognitive behavioral; and (4) integrative (Table 2). The most frequently used psychological approach was mindfulness, ACT, and self-compassion. General intervention activities and behavior change techniques, such as well-being tips and behavior change techniques to form habits, were adopted across psychological approaches and in most interventions (Textbox 1).

The intervention content was primarily developed by the study researchers and clinical psychologists (15/19, 79% of studies), some studies collaborated with companies or digital laboratories to develop the intervention (2/19, 11%), and some studies tested a preexisting intervention developed by a company (2/19, 11%).

**Table 2:** Description of intervention characteristics^a^.

Psychological approach underpinning the intervention	Activities or practices	Studies adopting the approach
Mindfulness, ACT^b^, and self-compassion	Meditation: awareness of inner experiences, present moment, and acceptance Overcoming obstacles in mindfulness meditation Body scan Increasing awareness through biofeedback Being mindful in daily life Loving-kindness meditation Compassionate journaling and breaks Self-kindness activities	[28,29,32,35,39,40,42]
Positive psychology	Gratitude (gratitude diary and letter) Positive future imagination Best possible self Counting blessings Random acts of kindness Replaying positive experiences Using strengths Savoring the moment Wearing a smile Brainstorming meaningfulness	[33,36-38,41]
Cognitive behavioral approach	Mood-related activities (eg, mood tracker, mood diary, and mood improvement activities) Challenging thoughts and behaviors Problem-solving Goal setting (SMART^c^ goals and planning) Committed actions Journaling	[26,37]
Integrative approach	A combination of intervention activities or practices of these psychological approaches	[25,27,30,31,34]

^a^For more detailed intervention description, refer to Multimedia Appendix 2.

^b^ACT: acceptance and commitment therapy.

^c^SMART: Specific, Measurable, Achievable, Relevant, and Time-bound.

**General psychological intervention components.**

**General intervention components adopted across interventions**

- Psychoeducation (eg, on emotions, needs, values, and mental illness)
- Support-seeking information
- Well-being tips

**Behavior change techniques adopted across interventions** [55]

- Habit formation
- Goal setting
- Action planning (eg, implementation intentions)
- Prompts or cues
- Self-monitoring of behavior or outcome of behavior
- Self-assessment of affective consequences
- Feedback on behavior
- Material or nonspecific reward

### Intervention Delivery

A total of 24 fully automated digital mental well-being interventions were included. The interventions were app based (n=10), web based (n=11), both app and web based (n=2), and SMS text message (n=1) interventions (Table 3).

**Table 3:** Intervention characteristics and dropout^a^.

Study, year	Participants randomized, N^b^	Intervention	Duration	Frequency	Mode of delivery	Dropout, n (%)^c^
Study 3 from Avey et al [25], 2022	102	Resilience intervention	10 wk	Weekly	App based	3 (2.9)
Bakker et al [26], 2018	226	Moodkit, Moodprism	30 d	Daily	App based	108 (47.8)
Brazier et al [27], 2022	279	Dear Doctor	10 mo	Fortnightly	SMS text message	126 (45.2)
Champion et al [28], 2018	74	Headspace	30 d	Daily	App based	12 (16.2)
Chung et al [29], 2021	427	Brief MBI^d^	6 wk	Weekly	Web based	280 (65.6)
Study 1 from Di Consiglio et al [30], 2021	24	Noibene	3 mo	4 times	Web based	0 (0)
Study 2 from Di Consiglio et al, [30], 2021	178	Noibene	None	None	Web based	119 (66.9)
Eisenstadt et al [31], 2021	115	Paradym	2 wk	Daily	App based	81 (70.4)
Gammer et al [32], 2020	206	Kindness For Mums Online	5 wk	Weekly	Web based	80 (38.8)
Liu et al [33], 2021	1000	Positive psychology intervention	1-3 d	Twice	Web based	132 (13.2)
Ly et al [34], 2017	30	Shim	2 wk	Daily	App based	3 (10)
Mak et al [35], 2018	2282	Mindfulness-based program and self-compassion program	28 d	Daily	App based and web based	1933 (84.7)
Manthey et al [36], 2016	666	Best possible self and gratitude	8 wk	Weekly	Web-based video	112 (16.8)
Mitchell et al [37], 2009	160	Strengths intervention and problem-solving intervention	3 wk	Daily	Web based	111 (77.6)
Neumeier et al [38], 2017	431	PERMA^e^ program and gratitude program	7 d	Daily	App based	128 (29.7)
Pheh et al [39], 2020	206	Brief MBI	1 d	Once	Web based	100 (48.5)
Schulte-Frankenfeld and Trautwein [40], 2021	99	Balloon	8 wk	Daily	App based	35 (35.4)
Shin et al [41], 2020	630	Gratitude writing	20 min	Once	Web based	49 (7.8)
Walsh et al [42], 2019	108	Wildflowers	3 wk	Daily	App based	22 (20.4)

^a^This table represents the general characteristics of the studies included in this systematic review. Only interventions of the studies that met the inclusion criteria are presented in this table.

^b^N denotes the number of participants randomized in the study, irrespective of whether people conducted baseline and follow-up assessments.

^c^Dropout rates are calculated from randomization to final assessment.

^d^MBI: mindfulness-based intervention.

^e^PERMA: Positive emotion, Engagement, Relationships, Meaning, Accomplishment.

### Intervention Duration, Frequency, and Timing

The participants were expected to use the intervention for substantially varied duration across interventions, ranging from 1 single session to 10 months, and there did not appear to be a clear end strategy across interventions. Most commonly, intervention use was recommended daily for up to 30 days, weekly for up to 8 weeks, and fortnightly for up to 10 months. Participants were often encouraged to use and access the intervention content for 5 to 15 minutes at a time, irrespective of the duration of the intervention.

### Level of Automation of Interventions

Access was generally automated with instant, sequential, or weekly access to content (Table 4). Most digital content was delivered in a standard way, and tailoring and dynamic delivery of content occurred in only 2 mental well-being interventions [34,42].

**Table 4:** Level of automation and engagement of intervention.

Study, year	Intervention	Frequency of content release	How access to intervention content was provided	Tailoring of content to improve or maintain engagement	Other digital intervention strategies to improve or maintain engagement	Actual engagement with intervention content^a^ (%)
Study 3 from Avey et al [25], 2022	Resilience intervention	Unknown	Unknown	None	None	Unknown
Bakker et al [26], 2018	Moodkit	Instant access	N/A^b^	N/A	None	Unknown
Bakker et al [26], 2018	Moodprism	Instant access	N/A	Feedback on mental well-being	None	Unknown
Brazier et al [27], 2022	Dear Doctor	Fortnightly	Automated text message	None	None	Unknown
Champion et al [28], 2018	Headspace	Sequential access	Automated access upon completion of step in the app	None	None	20.7
Chung et al [29], 2021	Brief MBI^c^	Fortnightly or weekly	Unknown	None	Notifying of new content	Unknown
Study 1 from Di Consiglio et al [30], 2021	Noibene	Instant access	N/A	None	None	100
Study 2 from Di Consiglio et al [30], 2021	Noibene	Instant access	N/A	None	None	Unknown
Eisenstadt et al [31], 2021	Paradym	Unknown	Unknown	None	Push notification	32.1
Gammer et al [32], 2020	Kindness for Mums Online	Weekly	Unknown	None	None	Unknown
Liu et al [33], 2021	Positive psychology intervention	Sequential access	Unknown	None	None	Unknown
Ly et al [34], 2017	Shim	Upon opening of app	Automated by digital conversational agent	On the basis of individual and external factors (eg, time of day)	None	126.5
Mak et al [35], 2018	Mindfulness-based Program	Weekly	Unknown	None	Sticker earning and alarm feature	29.5
Mak et al [35], 2018	Compassion-based program	Weekly	Unknown	None	Sticker earning and alarm feature	32.2
Manthey et al [36], 2016	Best possible self	Weekly	Automated email	None	None	Unknown
Manthey et al [36], 2016	Gratitude	Weekly	Automated email	None	None	Unknown
Mitchell et al [37], 2009	Strengths intervention	Instant access	N/A	None	Interactive features and automated email reminders	Unknown
Mitchell et al [37], 2009	Problem-solving intervention	Instant access	N/A	None	Interactive features and automated email reminders	Unknown
Neumeier et al [38], 2017	PERMA^d^ program	Sequential access	Automated access upon completion of step in program	None	None	Unknown
Neumeier et al [38], 2017	Gratitude program	Sequential access	Automated access upon completion of step in program	None	None	Unknown
Pheh et al [39], 2020	Brief MBI	Instant access	N/A	None	None	Unknown
Schulte-Frankenfeld and Trautwein [40], 2021	Balloon	Sequential access	Automated access upon completion of step in the app	None	A reminder was sent if a session was missed.	40.2
Shin et al [41], 2020	Gratitude writing	Instant access	N/A	None	None	100
Walsh et al [42], 2019	Wildflowers	Sequential access	Automated access upon completion of step in the app	On the basis of mood and stress levels recommendations were made for meditations	None	77.7

^a^Actual engagement with content is based on the requested frequency of engagement with the intervention (eg, daily for 2 wk=14 d=100%) compared with the actual frequency of engagement in the intervention (eg, on average, participants engaged with the intervention on 5 d=35.7%).

^b^N/A: not applicable.

^c^MBI: mindfulness-based intervention.

^d^PERMA: Positive emotion, Engagement, Relationships, Meaning, Accomplishment.

### Intervention Engagement

Overall, intervention engagement was suboptimal, below the required or recommended intervention engagement levels (Table 4). On average, participants engaged in 40.2% (median) of the recommended intervention sessions or days. Only few studies (3/19, 16%) contained optimal levels of engagement, engaging in the recommended intervention sessions or days or more [30,34,41].

Studies attempted to improve intervention engagement in a variety of different ways (Tables 2 and 4), including (1) sending automated email reminders or notifications to use the intervention, (2) increasing participant motivation (eg, increasing awareness of potential benefits and using in-app reward earning features), (3) increasing habit formation, and (4) tailoring intervention content based on external factors (such as time of day) or internal factors (such as suggestion of a specific activity based on someone’s mood).

Although caution should be used when interpreting the impact of these strategies on the engagement with the intervention because of the variety and inconsistency in reporting, preliminary results imply that tailored content improves engagement more than interventions that use reminders (habit formation and prompts) or sticker earning features (nonspecific rewards). Furthermore, it seems that interventions that require little engagement—engaging once or 4 times in the intervention in total [30,41]—also allow for more optimal intervention engagement. This is in line with studies showing that engagement was generally highest at the start of the intervention and decreased with time.

### Study Dropout and Attrition

Dropout occurred at any point throughout the study period when a participant failed to complete the research protocol associated with the digital intervention [56].

On average, there was a 37% dropout rate (mean), which ranged from 0% to 85% in the studies (Table 3). Strategies used to reduce study dropout included monetary incentives, the intervention being a mandatory element of university courses, and follow-up of participants by sending email reminders.

There were a range of findings across studies on the association between participants’ demographic characteristics and dropout. One study found that male participants were more likely to drop out [36], whereas others (2/19, 11%) found no difference [27,31]. Some studies (2/19, 11%) found that participants who remained in the study were older [35,38], although other studies (2/19, 11%) did not find this effect [31,36]. One study found that educational level was higher among participants who dropped out [35], whereas another study did not find this effect [38].

Several studies have compared whether baseline mental well-being was associated with dropout. Most studies (5/19, 26%) did not find any differences in baseline mental well-being levels between participants who did and did not drop out [27,29,32,35,36]. However, 1 study found that participants with lower mental well-being and higher levels of anxiety, depression, and distress were more likely to drop out [30], whereas another study found that participants with higher mental well-being and lower levels of anxiety, depression, and distress were more likely to drop out [31].

Few studies (2/19, 11%) excluded participants from their analysis (considered them to have dropped out) if they did not adhere with the intervention content at a minimum required level [37,42]; most studies (17/19, 89%) included participants with any level of intervention engagement.

### Outcomes

A variety of validated standardized questionnaires were used to measure mental well-being across studies, including the WHO 5 item mental well-being index and Warwick-Edinburgh Mental Well-Being Scale for mental well-being, Psychological Well-Being scale and Flourishing Scale for psychological well-being, and Satisfaction with Life Scale and Positive And Negative Affect Schedule for subjective well-being (Table 1). Nevertheless, the authors of 1 study created and validated their own mental well-being questionnaires, which included a combination of different measures. Although not included in this systematic review (as it is not considered the primary aim of mental well-being promotion), most studies (17/19, 89%) included additional outcome measures such as distress, depression, anxiety, and stress.

### RoB Assessments

Generally, the RoB of the included studies was considered to be high (Table 5). High levels of dropout and nonadherence led to a high RoB in domain 2 of Cochrane’s RoB-2.0 tool. This domain assesses RoB because of deviations from the intended interventions (effect of adhering to the intervention) and leads to high RoB, as the included studies did not appropriately account for intervention nonadherence in their analysis. For example, the Cochrane RoB-2.0 tool recommends using an instrumental variable analysis or inverse probability weighting to appropriately account for nonadherence; however, none of the included studies conducted these analyses.

**Table 5:** Bias assessment using Cochrane’s risk of bias (RoB) 2.0 tool^a^.

Study, year	Randomization process	Deviation from intended intervention	Missing outcome data	Measurement of outcome	Selection of the reported results	Overall RoB^b^
Study 3 from Avey et al [25], 2022	Some concerns^c^	High^d^	Low^e^	Some concerns	High	High
Bakker et al [26], 2018	Some concerns	High	Low	Some concerns	Some concerns	High
Brazier et al [27], 2022	Low	High	Low	Some concerns	Some concerns	High
Champion et al [28], 2018	Some concerns	High	Low	Some concerns	Low	High
Chung et al [29], 2021	High	High	High	High	Some concerns	High
Study 1 from Di Consiglio et al [30], 2021	Some concerns	High	Low	Some concerns	Some concerns	High
Gammer et al [32], 2020	Low	High	Low	Some concerns	Low	High
Liu et al [33], 2021	Some concerns	High	High	High	High	High
Ly et al [34], 2017	Low	High	Low	Some concerns	Some concerns	High
Mak et al [35], 2018	Low	High	Low	Some concerns	Some concerns	High
Manthey et al [36], 2016	Low	High	Some concerns	Low	Some concerns	High
Mitchell et al [37], 2009	Low	High	High	Low	Some concerns	High
Neumeier et al [38], 2017	Some concerns	High	High	High	Some concerns	High
Pheh et al [39], 2020	Some concerns	High	Some concerns	High	Some concerns	High
Schulte-Frankenfeld and Trautwein [40], 2021	Low	High	High	Some concerns	Some concerns	High
Shin et al [41], 2020	Low	Low	Low	High	Some concerns	High
Walsh et al [42], 2019	Low	High	Some concerns	High	Some concerns	High

^a^The National Institutes of Health bias assessment tool: before-after studies with no control group was used for study 2 from Di Consiglio et al [30], 2021 (overall RoB: high) and Eisenstadt et al [31], 2021 (overall RoB: high).

^b^The overall RoB judgement for that specific study.

^c^Some concerns: indicates that the authors considered there to be some concerns with the RoB for that study on that specific domain of the Cochrane RoB-2.0 tool.

^d^High: indicates that the authors considered there to be a high RoB for that study on that specific domain of the Cochrane RoB-2.0 tool.

^e^Low: indicates that the authors considered there to be a low RoB for that study on that specific domain of the Cochrane RoB-2.0 tool.

Furthermore, domain 4 in the RoB-2.0 tool, assessing RoB in measuring the outcome, led to a high RoB because of the nature of the research being fully automated and digital. Self-report measures were used to digitally assess mental well-being; however, participants were aware of the intervention they received when self-reporting their mental well-being scores, as most studies (11/19, 58%) included a waitlist control group. Although active controls account for this issue, these control interventions also contained high levels of dropout and therefore might not be appropriate as a control group [35].

A high RoB was also detected in studies because of the lack of general high-quality research practice. For example, several studies (7/19, 37%) did not provide any information regarding the randomization process, most studies did not preregister (12/19, 63%), and studies that did preregister (2/19, 11%) sometimes did not indicate their preintended analysis plan.

### Intervention Effects

All studies included fully automated digital mental well-being interventions in the general population and were therefore considered sufficiently homogeneous for a meta-analysis. Methodological homogeneity was also considered, which led to a comparison across RCTs only, as these were considered sufficiently homogeneous for a meta-analysis. Considering the incredibly high range of missing values, a meta-analysis based on ITT data was considered inappropriate; therefore, we conducted a meta-analysis based on PP data instead. Nevertheless, this increases the risk of underestimating or overestimating the real effect, which should be considered when interpreting the meta-result. Full PP data were available for a subset of 12 studies. A random-effect model was applied, as different measures were used to measure the same multidimensional construct *mental well-being*. Average effect estimates were computed for each study, with *negative affect* scores reversed to ensure that a higher score in each study indicated elevated levels of mental well-being. SMDs, 95% CIs, and 2-sided *P* values were calculated.

### Outlier

During data extraction, the negative affect score in the intervention group of 1 study [33] was flagged by both reviewers as unexpectedly high, and further information was sought to identify what could potentially explain this unusually large result. Normative data for negative affect was mean 14.8 (SD 5.4) [57]; however, the negative affect score in the waitlist control group in this study was mean 26.98 (SD 5.19). When exploring this data further, no methodological or clinical differences could reliably explain this result in our opinion. In addition, when included in the meta-analysis, CIs were entirely outside the range of any other study, and heterogeneity was incredibly high (92%; Multimedia Appendix 3). Removing this study from the meta-analysis reduced the overall heterogeneity from 92% to 50%. Therefore, the study was considered an outlier and was excluded from the meta-analysis.

### Main Effect

**Figure 2:**
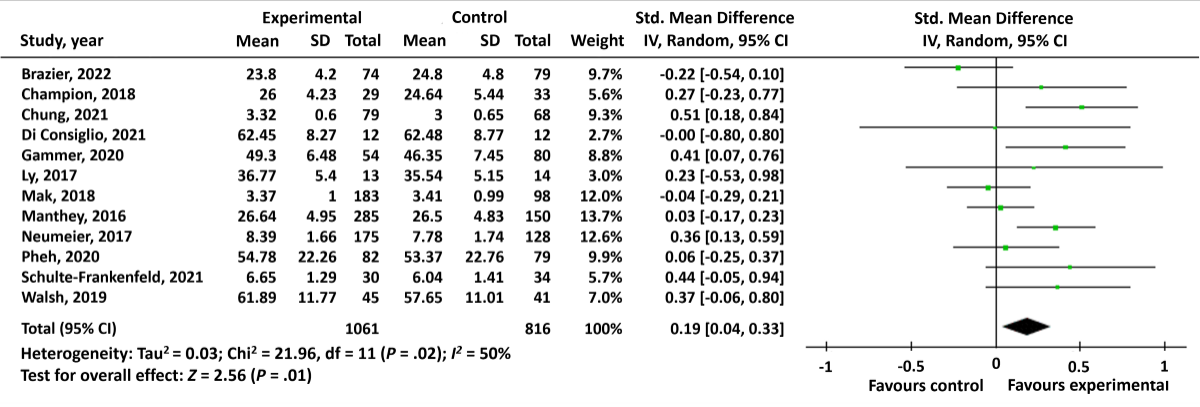
Per-protocol meta-analysis of fully automated digital interventions compared with control groups on mental well-being in the general population [27-30,32,34-36,38-40,42].

The pooled SMD, for the 12 trials, calculated using a random-effects model was 0.19 (95% CI 0.04-0.33; *P*=.01), indicating a small clinical effect in favor of digital mental well-being interventions (Figure 2). There was substantial heterogeneity (*I*^2^=50%).

### Sensitivity Analyses

As there was substantial heterogeneity (*I*^2^=50%), sensitivity analyses were performed to explore, interpret, and contextualize heterogeneity. First, intervention duration was explored using subgroups of interventions lasting up to 2 weeks (short), 2 to 6 weeks (medium), and >6 weeks (long).

A small significant effect was found for short interventions (SMD 0.24, 95% CI 0.04-0.45; *P*=.02) and medium interventions (SMD 0.29, 95% CI 0.05-0.52; *P*=.02); however, no effect was found for long interventions (SMD 0.02, 95% CI −0.22 to 0.26; Figure S1 in Multimedia Appendix 4). No significant levels of heterogeneity were found in any of the subgroups (all *P*>.05), and the subgroups substantially reduced the overall level of heterogeneity (*I*^2^=28.6%).

Another sensitivity analysis was performed to explore methodological heterogeneity across studies based on the comparator. We argue that placebo controls are not feasible in psychological interventions, considering the difficulty in isolating intervention components in psychological interventions [58]. Therefore, we grouped placebo controls under active controls in this review. A small significant effect was found in studies using a waitlist control as a comparator (SMD 0.28, 95% CI 0.07-0.50; *P*=.008), but no significant effect was found in studies using a placebo or active control as a comparator (SMD 0.05, 95% CI −0.08 to 0.18; *P*=.49; Figure S2 in Multimedia Appendix 4). No significant levels of heterogeneity were present in either of the 2 subgroups (all *P*>.05), although substantial heterogeneity remained in studies that used a waitlist control comparator (*I*^2^=53%).

Finally, a sensitivity analysis was performed based on the outcomes of mental well-being, psychological well-being, and subjective well-being. A small significant effect was found on subjective well-being (SMD 0.23, 95% CI 0.04-0.42; *P*=.02). However, no significant effect was found on mental well-being (SMD 0.14, 95% CI −0.12 to 0.40; *P*=.31) or psychological well-being (SMD 0.26, 95% CI −0.08 to 0.59; *P*=.14; Figure S3 in Multimedia Appendix 4). Despite reducing heterogeneity in subjective well-being and psychological well-being, substantial heterogeneity was found in mental well-being (*I*^2^=72%).

### Reporting Bias

**Figure 3:**
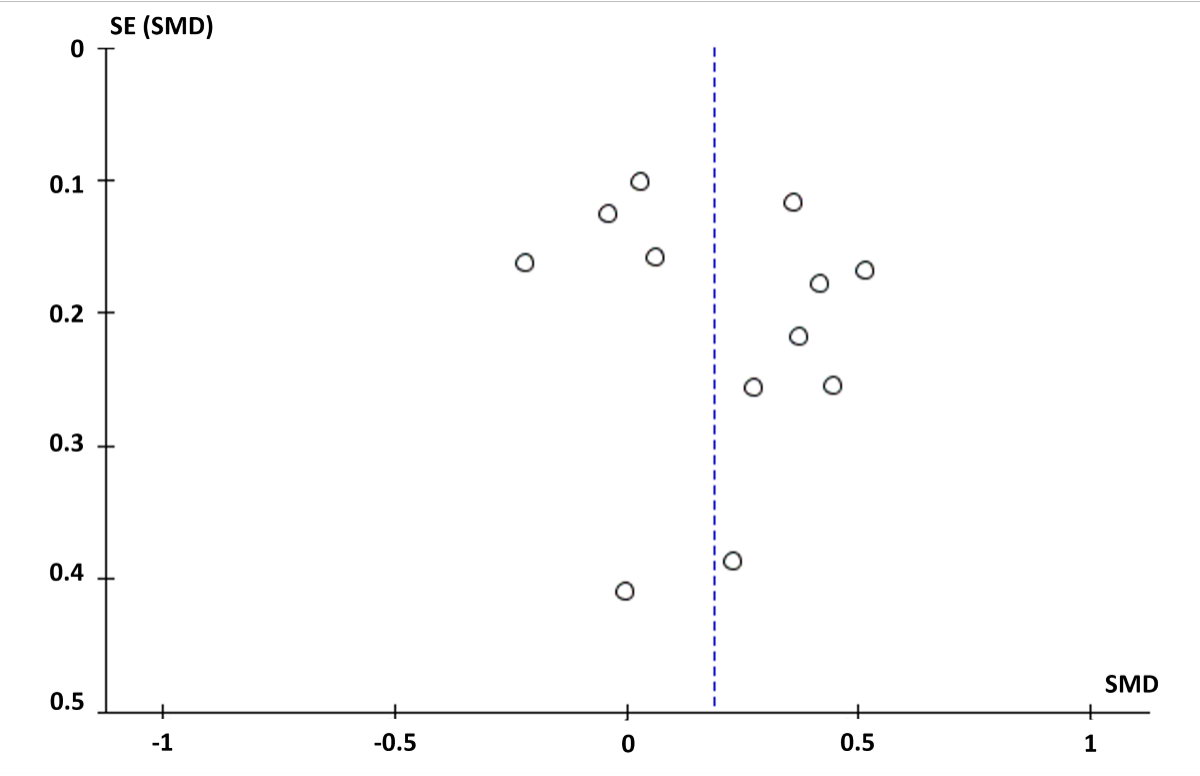
Funnel plot. Asymmetrical plot due to the presence of publication bias or low methodological quality studies. The funnel plot only represents studies that were included in the main per-protocol meta-analysis. SMD: standardized mean difference.

Visual inspection of the funnel plot, which appeared asymmetrical, indicated evidence of reporting bias (Figure 3). Few smaller studies were found, and larger random variation would be expected within smaller studies; this is potentially because of a publication bias, although other aspects such as heterogeneity can also cause asymmetrical funnel plots.

### Certainty of Body of Evidence (Grading of Recommendations, Assessment, Development, and Evaluations)

The certainty of the body of evidence was assessed using Grading of Recommendations, Assessment, Development, and Evaluations [59]. The evidence was downgraded because of high RoB (effect of adhering to the intervention; Table 5), inconsistency (heterogeneity was considered substantial; Figure 2), imprecision (wide CIs and insufficiently small sample sizes were observed; Figure 2), and publication bias (visual asymmetry in the funnel plot; Figure 3). Thus, we consider a very low confidence in the quality of evidence of the main PP meta-effect (Figure 2), meaning that we are very uncertain about the estimate of the effect.

### Subgroup Analysis

An a priori subgroup analysis was planned to detect the effects of digital mental well-being interventions across individual differences (eg, age, sex, and educational level). Nevertheless, insufficient data were available for a meaningful comparison to be made.

**Figure 4:**
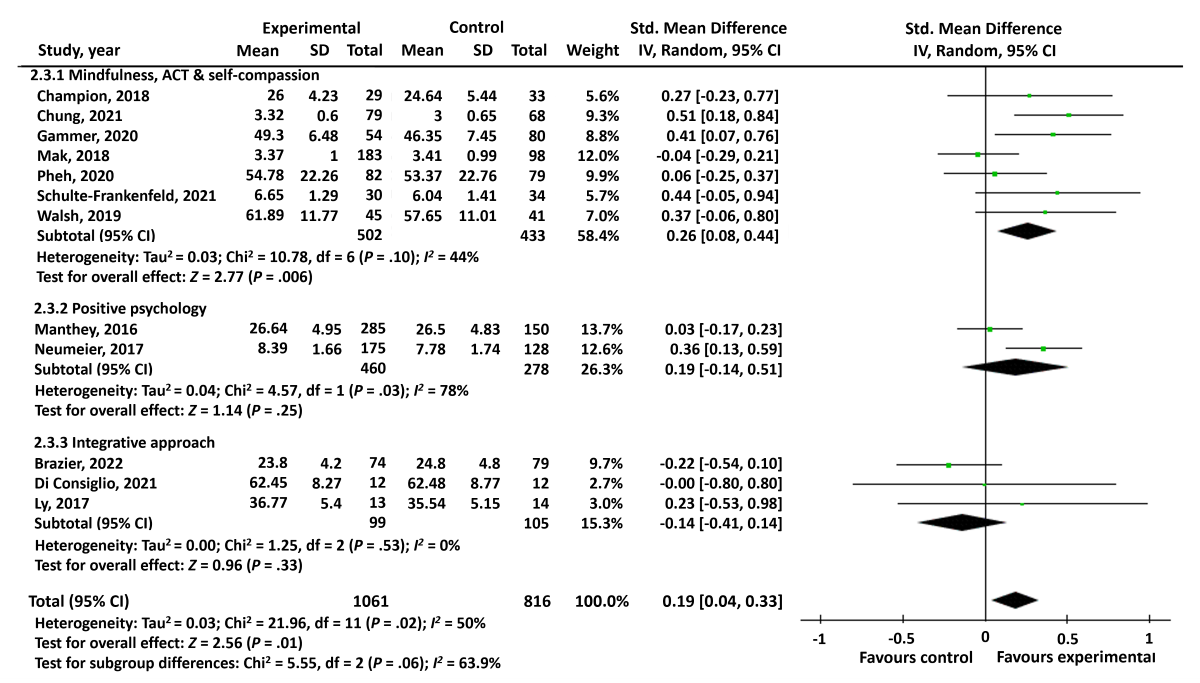
Subgroup analysis of different psychological approaches to promote mental well-being [27-30,32,34-36,38-40,42].

Another a priori subgroup analysis was planned to identify the effectiveness across psychological approaches. Mindfulness, ACT, and self-compassion interventions were the most common. A total of 7 studies were included in this subgroup. A small significant effect was found for fully automated digital mindfulness, ACT, and self-compassion interventions to promote mental well-being in the general population (SMD 0.26, 95% CI 0.08-0.44; *P*=.006), with moderate levels of heterogeneity (*I*^2^=44%; Figure 4). The positive psychology intervention subgroup only included 2 studies, and there were significant levels of heterogeneity (*P*=.03; *I*^2^=78%). Studies investigating CBT-based interventions did not contain any PP data and could therefore not be included as a subgroup in the analysis. The final subgroup included an integrative approach; 3 studies contained sufficient PP data to be included. There was no significant level of heterogeneity in this subgroup (*P*=.53; *I*^2^=0%); however, integrative approaches did not have a significant effect on mental well-being in the general population (*P*=.33).

Overall, no significant subgroup difference was found when comparing the effects of mindfulness, ACT, self-compassion, positive psychology, and integrative interventions on mental well-being (*P*=.06).

## Discussion

### Main Effect

The aim of this systematic review and meta-analysis was to understand the effectiveness of fully automated digital interventions in promoting mental well-being in the general population. We evaluated 24 fully automated digital mental well-being interventions lasting from a single session to 10 months, with daily, weekly, and biweekly delivery. After the intervention, we found a small significant effect of fully automated digital mental well-being interventions compared with control groups on mental well-being in the general population.

The effect found in this meta-analysis of fully automated digital interventions (SMD 0.19) was smaller than the effect found in previous meta-analyses of nonautomated mental well-being interventions (effect sizes ranging between 0.26 and 0.42) [7,15,16]. This could highlight the importance of nonspecific psychological factors, such as the therapeutic relationship and social support, in the effectiveness of these psychological interventions. In contrast, this could also indicate the importance of social support in the adherence to mental well-being interventions. Previous research found that improved adherence was linked to better mental well-being outcomes and that adherence tended to be higher in nonautomated interventions [18,56]. As suboptimal intervention adherence was observed in this review, with average engagement in 40% of the intervention content, it is likely that the reported effectiveness in this review is an underestimation of the potential effectiveness of fully automated digital interventions that could be achieved when reaching optimal levels of engagement (the level of engagement recommended by the researchers). Nevertheless, the recommended engagement levels differed tremendously between studies, and studies lacked a clear end strategy.

### Exploratory Findings

We found that short (<2 wk) and medium (<6 wk) interventions were effective in promoting mental well-being in the general population but long (>6 wk) interventions were not. This could be further related to intervention adherence, as (in line with previous research findings) intervention adherence reduced with time [56]. It does appear that the optimal intervention duration may also depend on the outcome that is being targeted. Research has found that short interventions led to a greater effect on subjective well-being, whereas long interventions had a greater effect on psychological well-being [60]. As most studies (9/19, 47%) in this review included a subjective well-being outcome, this might explain why shorter interventions were found to be effective in this review.

In contrast to prior research, our exploratory analysis showed no significant effect on general mental well-being outcomes (eg, Warwick-Edinburgh Mental Well-Being Scale) [7,15]. Measures of general mental well-being might lack the sensitivity to detect subtle changes occurring within the general population. This could be attributed to the concise nature of mental well-being measures, which encompass both subjective and psychological aspects [47]. Previous research includes a clinical population alongside a general population and nonautomated interventions alongside fully automated digital mental well-being interventions [7,14,15]. Both these factors increase the effectiveness of mental well-being interventions, which could lead to a sufficiently large effect to detect using a general mental well-being measure.

Furthermore, we found a small significant effect when comparing a fully automated digital mental well-being intervention with a waitlist control group, although no significant effect was found when comparing it with an active or placebo control group. The effect when compared with an active and placebo control is expected to be smaller than the effect when compared with a passive control [61]. This indicates that the effects of mental well-being interventions and other psychological interventions (eg, active control) on mental well-being do not currently differ.

### Subgroup Effects

It was not possible to analyze the effects of digital mental well-being promotion across population subgroups (based on age, sex, socioeconomic status, and educational level) because of a lack of studies reporting these results separately.

Nevertheless, studies did provide exploratory findings on the relationship between individual differences and dropout in fully automated digital mental well-being interventions. These exploratory findings indicated largely conflicting evidence on whether and how individual differences were related to dropout, which is in line with previous research findings [56].

A subgroup analysis comparing psychological approaches adopted in fully automated digital mental well-being interventions indicated a small significant effect of fully automated digital mindfulness-, ACT-, and compassion-based interventions on mental well-being in the general population, with most studies (7/19, 37%) adopting this psychological approach. The effectiveness of fully automated digital positive psychology and CBT-based approaches remains largely unknown. A potential explanation for this is the large focus of CBT-based interventions on symptom reduction rather than on mental well-being improvement [62]. Furthermore, positive psychology interventions have been criticized recently because of the limited ability of studies to replicate positive psychology results [63], potentially leading to fewer studies investigating positive psychology interventions.

Finally, although several studies (6/19, 32%) have adopted an integrative approach, we did not find an effect of fully automated digital integrative approaches on mental well-being in the general population. This contradicts previous meta-analytic findings that found a significant effect of multitheoretical interventions on mental well-being in the general population [7]. Nevertheless, previous meta-analysis also found a smaller effect for multitheoretical interventions compared with MBIs [7], indicating that these interventions might generally be less effective. This might explain why no effect of integrative approaches was found in fully automated digital interventions.

### Limitations

Several methodological limitations should be recognized; however, as they could have impacted the findings of this systematic review. First, the specific search terms adopted in this systematic review limit the findings. Although searches should aim to be as comprehensive as possible, it is necessary to balance sensitivity and specificity when conducting searches [64]. The specificity adopted in this systematic review may not have allowed the searches to be comprehensive, as the literature uses many different terms to describe fully automated digital mental well-being interventions. Second, the inclusion criteria in this systematic review are ambiguous and require judgement [64]. This subjectivity could lead to lower reproducibility of the findings and random errors and biases [65]. Finally, the review adopts an exclusive focus on mental well-being (which includes both subjective and psychological well-being). Although improving mental well-being could be considered the primary aim of digital mental well-being promotion [10], the exclusive focus on mental well-being does not allow the review to provide insights into indirect positive or negative intervention effects.

In addition to methodological limitations, we observed several limitations of the included studies that lowered confidence in the quality of evidence (Grading of Recommendations, Assessment, Development, and Evaluations). We saw a high RoB in the included studies because of the following reasons: (1) missing outcome data—although it is unknown what impact the dropout has on the overall effect (eg, underestimation or overestimation) as reasons for dropout remain largely unknown; (2) the effect of adherence—suboptimal adherence might lead to an underestimation of the effectiveness; and (3) measurement of the outcome—because of the use of self-report measures while participants are aware of their allocated intervention, potentially leading to overestimation of the effectiveness. In addition, we also found a lack of general high-quality research practice in studies. Several studies were underpowered, did not provide sufficient information regarding randomization, and did not preregister or contain a prespecified analysis plan.

Furthermore, we detected a publication bias of the studies included in the meta-analysis. This publication bias indicated that smaller studies with a larger random variation were largely missing, perhaps because they were less likely to be published.

Finally, the fully automated digital mental well-being interventions were primarily delivered in a Western context and typically included a sample of participants who were highly educated and female, which might limit the generalizability of the findings. In particular, there is evidence that females and highly educated individuals might engage with and therefore benefit from these interventions differently.

### Recommendations for Future Research

The systematic review findings lead to several implications for future research. First, future research should aim to focus in more detail on supporting engagement and reducing dropout in fully automated digital mental well-being interventions—by understanding the impact of behavioral strategies, such as habit formation and nonspecific rewards [55], and also by examining what is considered *effective engagement*—the target level of intervention engagement needed for change [66]. This will allow for evidence-based recommendations of the level of intervention engagement in future research and practice and for studies to adopt effective end strategies.

Second, future research should look to understand how automated digital interventions can be *tailored* to deliver relevant content according to the preferences of the user and whether tailoring is necessary to ensure intervention effectiveness and whether acceptability can be ensured across different populations (eg, Western vs non-Western) and intervention types (eg, positive psychology vs mindfulness and ACT).

Finally, we recommend that future research strictly follows high-quality research recommendations, such as the CONSORT (Consolidated Standards of Reporting Trials) statement [67], when investigating fully automated digital mental well-being interventions to allow for higher confidence in the quality of the evidence.

### Conclusions

Overall, this review provides a novel insight into the effectiveness of fully automated digital mental well-being interventions in the general population. It shows that fully automated digital mental well-being interventions can effectively promote mental well-being in the general population (particularly when adopting a mindfulness-, ACT-, and self-compassion–based approach), despite low levels of intervention adherence and high study dropout.

## Appendix

Multimedia Appendix 1 Search strategy per database.

Multimedia Appendix 2 Table with detailed intervention description.

Multimedia Appendix 3 Main per-protocol analysis including outlier [27-30,32-36,38-40,42].

Multimedia Appendix 4 Exploring heterogeneity [27-30,32,34-36,38-40,42].

Multimedia Appendix 5 PRISMA Checklist.

## Abbreviations

ACT: acceptance and commitment therapy
CBT: cognitive behavioral therapy
CONSORT: Consolidated Standards of Reporting Trials
ITT: intention to treat
MBI: mindfulness-based intervention
PP: per-protocol
PRISMA: Preferred Reporting Items for Systematic Reviews and Meta-Analyses
RCT: randomized controlled trial
RoB: risk of bias
SMD: standardized mean difference
WHO: World Health Organization

## Data Availability

For the purpose of open access, the author has applied a Creative Commons Attribution (CC-BY) license. Data supporting this study are openly available from UK Data Service.
