# Supplementary material for: The Effectiveness of Fully Automated Digital Interventions in Promoting Mental Well-Being in the General Population: Systematic Review and Meta-Analysis
Source: JMIR Ment Health. 2023 Oct 19;10:e44658. doi: 10.2196/44658 (PMC10623223; doi:10.2196/44658)
Supplement: Multimedia Appendix 1 [file mental_v10i1e44658_app1.docx]

**Multimedia Appendix 1. Search strategy per database**

*Medline*

("mental well being"[Title/Abstract] OR "mental wellbeing"[Title/Abstract] OR "psychological well being"[Title/Abstract] OR "psychological wellbeing"[Title/Abstract] OR "subjective wellbeing"[Title/Abstract] OR "subjective well being"[Title/Abstract]) AND ("digital*"[Title/Abstract] OR “online”[Title/Abstract] OR "internet"[Title/Abstract] OR "web-based"[Title/Abstract] OR "app"[Title/Abstract] OR "apps"[Title/Abstract] OR "smartphone application*"[Title/Abstract] OR "mobile application*"[Title/Abstract])

*Web of Science*

(TS=("mental wellbeing") OR TS=("mental well being") OR TS=("psychological wellbeing") OR TS=("psychological well being") OR TS=("subjective wellbeing") OR TS=("subjective well being")) AND (TS=("digital*") OR TS=("online") OR TS=("web-based") OR TS=("internet") OR TS=("app") OR TS=("apps") OR TS=("smartphone application*") OR TS=("mobile application*”))

*Cochrane*

("mental well being" OR "mental wellbeing" OR "psychological well being" OR "psychological wellbeing" OR "subjective well being" OR "subjective wellbeing" in Title Abstract Keyword) AND ("Digital*" OR "online" OR "web-based" OR "Internet" OR "app" OR "apps" OR "smartphone application*" OR “mobile application*” in Title Abstract Keyword)

*PsychINFO & PsychEXTRA*

((**Keywords**: ("mental wellbeing")) *OR* (**title**: ("mental wellbeing")) *OR* (**abstract**: ("mental wellbeing")) *OR* (**Keywords**: ("mental well being")) *OR* (**title**: ("mental well being")) *OR* (**abstract**: ("mental well being")) OR (**Keywords**: ("psychological wellbeing")) *OR* (**title**: ("psychological wellbeing")) *OR* (**abstract**: ("psychological wellbeing")) *OR* (**Keywords**: ("psychological well being")) *OR* (**title**: ("psychological well being")) *OR* (**abstract**: ("psychological well being")) *OR* (**Keywords**: ("subjective wellbeing")) *OR* (**title**: ("subjective wellbeing")) *OR* (**abstract**: ("subjective wellbeing")) *OR* (**Keywords**: ("subjective well being")) *OR* (**title**: ("subjective well being")) *OR* (**abstract**: ("subjective well being"))) *AND* ((**abstract**: (“digital*”)) *OR* (**abstract**: (“online”)) *OR* (**abstract**: (“web-based”)) *OR* (**abstract**: (“internet”)) *OR* (**abstract**: (“app”)) *OR* (**abstract**: (“apps”)) *OR* (**abstract**: (“smartphone application*”)) *OR* (**abstract**: (“mobile application*”)) *OR* (**title**: (“digital*”)) *OR* (**title**: (“online”)) *OR* (**title**: (“web-based”)) *OR* (**title**: (“internet”)) *OR* (**title**: (“app”)) *OR* (**title**: (“apps”)) *OR* (**title**: (“smartphone application*”)) *OR* (**title**: (“mobile application*”)) *OR* (**Keywords**: (“digital*”)) *OR* (**Keywords**: (“online”)) *OR* (**Keywords**: (“web-based”)) *OR* (**Keywords**: (“internet”)) *OR* (**Keywords**: (“app”)) *OR* (**Keywords**: (“apps”)) *OR* (**Keywords**: (“smartphone application*”)) *OR* (**Keywords**: (“mobile application*”)))

*Scopus*

( TITLE-ABS-KEY ( "mental well being" OR "mental wellbeing" OR "psychological well being" OR "psychological wellbeing" OR "subjective well being" OR "subjective wellbeing" ) ) AND ( TITLE-ABS-KEY ( "Digital*" OR "online" OR "web-based" OR "Internet" OR "app" OR "apps" OR "smartphone application*" OR "mobile application*" ) )

*ACM Digital*

[[Title: "mental well being"] OR [Title: "mental wellbeing"] OR [Title: "psychological well being"] OR [Title: "psychological wellbeing"] OR [Title: "subjective well being"] OR [Title: "subjective wellbeing"] OR [Abstract: "mental well being"] OR [Abstract: "mental wellbeing"] OR [Abstract: "psychological well being"] OR [Abstract: "psychological wellbeing"] OR [Abstract: "subjective well being"] OR [Abstract: "subjective wellbeing"] OR [Keywords: "mental well being"] OR [Keywords: "mental wellbeing"] OR [Keywords: "psychological well being"] OR [Keywords: "psychological wellbeing"] OR [Keywords: "subjective well being"] OR [Keywords: "subjective wellbeing"]] AND [[Title: "digital*"] OR [Title: "online"] OR [Title: "web-based"] OR [Title: "internet"] OR [Title: "app"] OR [Title: "apps"] OR [Title: "smartphone application*"] OR [Title: "mobile application*"] OR [Abstract: "digital*"] OR [Abstract: "online"] OR [Abstract: "web-based"] OR [Abstract: "internet"] OR [Abstract: "app"] OR [Abstract: "apps"] OR [Abstract: "smartphone application*"] OR [Abstract: "mobile application*"] OR [Keywords: "digital*"] OR [Keywords: "online"] OR [Keywords: "web-based"] OR [Keywords: "internet"] OR [Keywords: "app"] OR [Keywords: "apps"] OR [Keywords: "smartphone application*"] OR [Keywords: "mobile application*"]]
