# Supplementary material for: The Effectiveness of Fully Automated Digital Interventions in Promoting Mental Well-Being in the General Population: Systematic Review and Meta-Analysis
Source: JMIR Ment Health. 2023 Oct 19;10:e44658. doi: 10.2196/44658 (PMC10623223; doi:10.2196/44658)
Supplement: Multimedia Appendix 2 [file mental_v10i1e44658_app2.docx]

**Multimedia Appendix 2. Table with detailed intervention description**

*Detailed intervention description*

| Study | Intervention name | Intervention components |
| --- | --- | --- |
| Bakker, 2018 | MoodKit | - Thought checker *– feel better by changing how you think* - Mood tracker - Moodkit journal - Mood improvement activities - Psychoeducation |
|  | MoodPrism | - Mood tracker with feedback - Mood diary – *displays your tracked mood over time* - Links to mental health resources |
| Brazier, 2022 | ‘Dear Doctor’ | - Expressing gratitude to others - Counting blessings *– reflecting on 3 good things daily* - Receiving gratitude through intervention - Connection to purpose *– reflect on daily tasks and its wider purpose* - Self-efficacy – - reflect on achievements - reflect on progress - providing means to improve self-control during covid-19 - Mindfulness links - Self compassion – ‘plan to do something positive for yourself’ - Reducing barriers to support seeking - Sharing resources - Quotes: ‘it’s ok not to be ok’ - Social support - Discuss work related difficulties - Ask colleague about their well-being - Consider who could support you when you’re struggling - Professional community - Encourage discussion at work - Promote people checking in with each other - Share positive and negative experiences - Express gratitude to colleagues - Planning prompt - Implementation intentions (if … then plans) for difficult future situations - Plan who to approach if needed - Routine for switching off after work - Find time for self-compassion |
| Champion, 2018 | Headspace | Specific exercise components:   - Day 1-10 Basics of mindfulness breathing meditation - Awareness and acceptance of inner experiences (mindset, mood, feelings, thoughts, physically) - Day 11-20 Overcoming obstacles during practice: - Restless mind - Lethargic mind - Bored mind - Physical discomfort - Lost in (pleasant) daydreams - Day 21-30 Integrating mindfulness in everyday life - Practice awareness throughout the day - Mindfulness meditation throughout the day - Mindfulness during daily activities & movement   General intervention components:   - Identifying/increasing motivation - Impact of practice on everyday life and social relations - Praise during exercises - Building a routine - Prioritise practice - Reminders - Plan - Track progress |
| Chung, 2021 | Brief MBI | - Focus on present moment and awareness of inner experiences - Focus awareness on: - Sounds - Sights - Breathing - Focus on the connection and awareness of current experiences |
| Di Consiglio, 2021 – Study 1 | NoiBene | Commitment and Motivation:   - The concept of well-being is explained according with the PERMA model (Seligman et al., 2005). - Commitment and motivation are elicited in students by asking them to schedule a timetable of future access to the platform.   Self-awareness:   - Psychoeducation about emotions, needs and values. - Students are asked to use a personal diary, inspired by Ellis’ ABC technique (1995), designed to report one’s own emotive episodes. Then, some exercises are proposed to help students identify their values system.   Social Skills:   - Psychoeducation about the passive, aggressive and assertive style of communication. According with the NCV model (Rosenberg & Chopra, 2015), the various steps to develop an assertive and emphatic communication are presented. Psychoeducation about gratitude. - Students are asked to complete some exercise and self-monitoring tool to identify their predominant communicative style. Then, for each step of NCV, there is a self-monitoring tool to fully understand the operating modes. Lastly, some exercise to increase gratitude are proposed (i.e. the gratitude diary and the gratitude letter, Seligman, 2005).   Self-realization:   - Introduction of the SMART model (Wade, 2009) to reach personal goals. - Students have to set a goal following the SMART model. Students are encouraged to set goals in accordance with their values (previously expose in the Self-awareness module). |
| Di Consiglio, 2021 – Study 2 | NoiBene | Commitment and Motivation:   - The concept of well-being is explained according with the PERMA model (Seligman et al., 2005). - Commitment and motivation are elicited in students by asking them to schedule a timetable of future access to the platform.   Self-awareness:   - Psychoeducation about emotions, needs and values. - Students are asked to use a personal diary, inspired by Ellis’ ABC technique (1995), designed to report one’s own emotive episodes. Then, some exercises are proposed to help students identify their values system.   Psychological Bugs:   - Introduction to the concept of Psychological bugs (e.g. mental processes and attitudes that contribute to emotional suffering and relationship problems such as repetitive thinking, (rumination, worry and self-criticism; Ehring & Watkins, 2008) perfectionism (Shafran & Mansell, 2001), and experiential avoidance (Chawla & Ostafin, 2007). - Students have to answer a series of questionnaires to identify the presence of psychological bugs. Then students are directed towards the modules that are more suitable to their vulnerabilities.   Repetitive thinking:   - Psychoeducation about rumination, worry and self-criticism. - Students are asked to use a personal diary to monitor their repetitive thinking behavior. Students are encouraged to follow some techniques to challenge such thoughts (i.e. self-compassion techniques).   Perfectionism:   - Psychoeducation about perfectionism. - Exercise to identify personal high standards and the positive and negative aspects for being a perfectionist. Students are encouraged to follow some techniques to challenge perfectionistic thoughts and behavior.   Avoidance:   - Psychoeducation about avoidance. - Students are asked to use a personal diary, inspired by Ellis’ ABC technique (1995), focusing on the situations that they tend to avoid. Students are encouraged to follow some techniques to challenge avoidance.   Social Skills:   - Psychoeducation about the passive, aggressive and assertive style of communication. According with the NCV model (Rosenberg & Chopra, 2015), the various steps to develop an assertive and emphatic communication are presented. Psychoeducation about gratitude. - Students are asked to complete some exercise and self-monitoring tool to identify their predominant communicative style. Then, for each step of NCV, there is a self-monitoring tool to fully understand the operating modes. Lastly, some exercise to increase gratitude are proposed (i.e. the gratitude diary and the gratitude letter, Seligman, 2005).   Self-realization:   - Introduction of the SMART model (Wade, 2009) to reach personal goals. - Students have to set a goal following the SMART model. Students are encouraged to set goals in accordance with their values (previously expose in the Self-awareness module).   Psychological First Aid:   - Psychoeducation about loneliness, rejection, failure, emotional loss. Psychoeducation about anxiety and depression. - Some useful tools and strategies to help students to face with rejection, loneliness, failure, and emotional loss are presented. |
| Eisenstadt, 2021 | Paradym | 5 overarching themes: aware, success, love, identity, body   - Psychoeducation - Journaling - Reflect on emotional patterns - Daily reflections - Increasing awareness of the benefits of exercises |
| Gammer, 2020 | Kindness For Mums Online (KFMO) | - Psychoeducation on self-kindness - Activity with intention of self-kindness and notice reactions - Psychoeducation on being self-critical - What would you say to a friend? - Highlight situations that might lead to being self-critical and how to be self-kind - Choose object in certain situation which would remind of self-kindness - Becoming mindful of experience - Psychoeducation on emotional awareness and ambivalence - Mindfulness of natural object - Post-it notes with kindness - Reflection on emotions related to other people’s judgement - Reflection on changing relationships - Do something kind for someone else - Psychoeducation on expectations vs. reality - Reflect on something positive in the past - Identity acceptance - Recognise own milestones |
| Liu, 2021 | Positive Psychology Intervention (PPI) | - Positive future imagination - Gratitude |
| Ly, 2017 | Shim | PP:   - Expressing gratitude - Practicing kindness - Engaging in enjoyable activities - Replaying positive experiences   CBT:   - Present moment awareness - Valued directions - Committed actions   Reflect, learn and practice strategies and behaviours |
| Mak, 2018 | Mindfulness-based program | - Mood tracker - Well-being tips - Body scan - Mindful breathing - Mindful eating - Mindful walking - 3-min breathing space - Thought distancing - Psychoeducation of mindfulness and its difficulties |
|  | Self-compassion program | - Mood tracker - Well-being tips - Compassionate body scan - Affectionate breathing - Loving-kindness meditation for beginners - Compassionate walking - Soften-allow-soothe - Self-compassion break - Self-compassion journaling - Psychoeducation of self-compassion and mental health |
| Manthey, 2016 | Best possible self | Best possible self (imagine what future life would ideally look like) focused on:   - social relations, partner, hobbies and personal interests, family, friendship, clubs, networks, groups and community, physical and mental health, job, career and free topic. |
|  | Gratitude | - Reflect on things you’re grateful for each week |
| Mitchell, 2009 | Strengths intervention | - Identify and prioritise strengths from list of 24 signature strengths - Share with a friend - Reflect - Practice signature strength - Track progress with diary - Reflect on progress |
|  | Problem solving intervention | - 6-step approach to problem solving - Share learned experience with friend/family - Reflect - Apply approach to problem solving to a real-life problem - Track progress in diary |
| Neumeier, 2017 | PERMA programme | - Practicing gratitude - Savouring the moment - You at your best (reflecting on strengths) - Random acts of kindness - Visualising your best possible self - Wearing a smile - Brainstorming meaningfulness |
|  | Gratitude programme | - Gratitude exercises |
| Pheh, 2020 | Brief Mindfulness-Based Intervention (MBI) | - Recognising cognitive and affective experiences - 3 things you can do to make yourself feel better - Reflect on privileges and benefits - 3 things you can do for yourself and others |
| Schulte-Frankenfeld, 2021 | Balloon | - Mindfulness meditation - Present moment awareness - Observing thoughts and feelings - Compassion - Care towards oneself |
| Walsh, 2019 | Wildflowers | - Meditations - Guided meditations suggested based on mood and stress levels - Mindful breathing - Body scans - Open monitoring practices - Lessons and information about benefits of mindfulness - Biofeedback: mood and stress levels are tracked and provided as feedback to create awareness of physiological and psychological benefits of mindfulness |
| Avey, 2022; study 3 | Resilience intervention | - Psychoeducation on resilience - Recognising emotional and behavioural responses in everyday life - Practicing and building resilience |
| Shin, 2020 | Gratitude writing | - Writing a gratitude letter to a parent |
