# Supplementary material for: The Effectiveness of Fully Automated Digital Interventions in Promoting Mental Well-Being in the General Population: Systematic Review and Meta-Analysis
Source: JMIR Ment Health. 2023 Oct 19;10:e44658. doi: 10.2196/44658 (PMC10623223; doi:10.2196/44658)

**Multimedia Appendix 3. Main PP analysis including outlier**

*Main PP analysis including outlier Liu et al. (2021)*


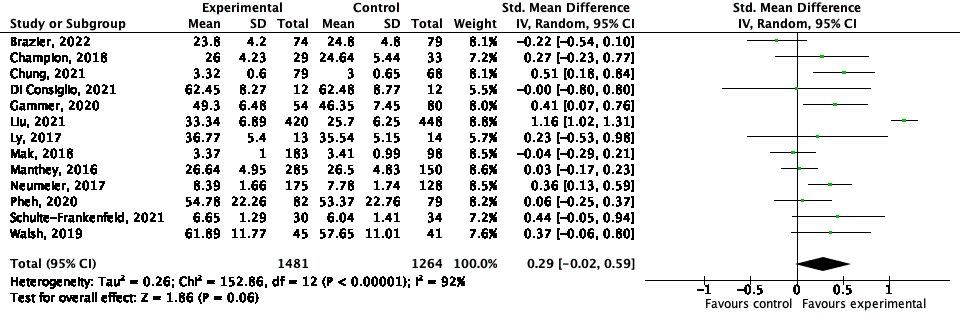

Supplement: Multimedia Appendix 3 [file mental_v10i1e44658_app3.docx]
