# Supplementary material for: The Effectiveness of Fully Automated Digital Interventions in Promoting Mental Well-Being in the General Population: Systematic Review and Meta-Analysis
Source: JMIR Ment Health. 2023 Oct 19;10:e44658. doi: 10.2196/44658 (PMC10623223; doi:10.2196/44658)

**Multimedia Appendix 4. Exploring heterogeneity**

**Figure S1.**

*Exploring methodological heterogeneity across studies: subgroup analysis of intervention duration*


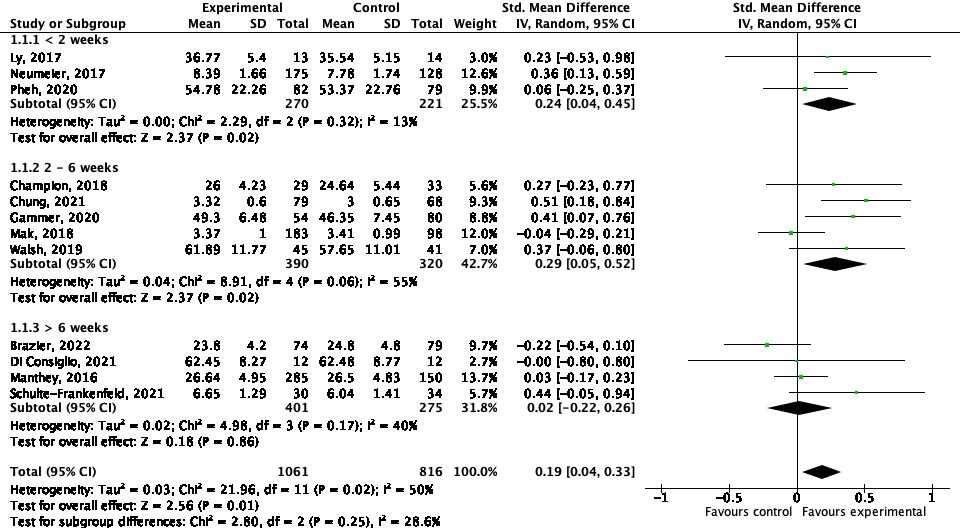


**Figure S2.**

*Methodological heterogeneity based on comparator*


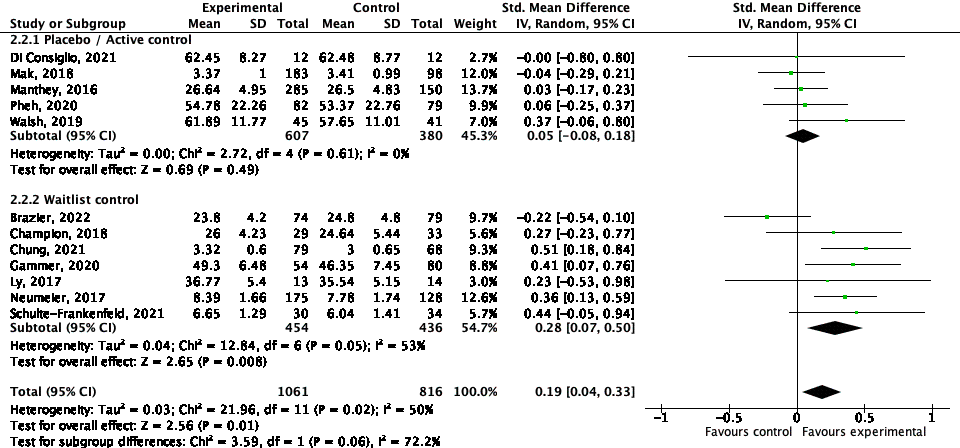


**Figure S3.**

*Clinical heterogeneity based on outcome*


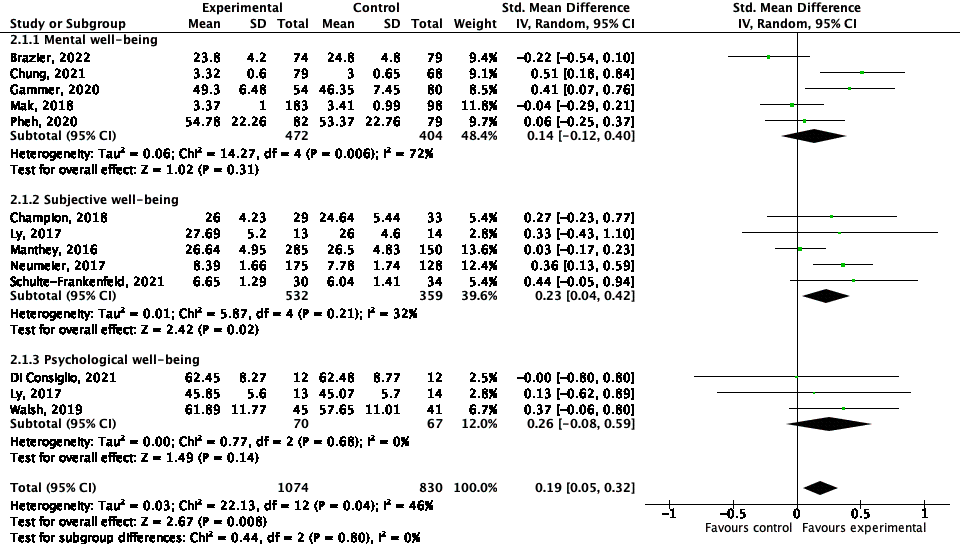

Supplement: Multimedia Appendix 4 [file mental_v10i1e44658_app4.docx]
